# A generalized framework unifying image registration and respiratory motion models and incorporating image reconstruction, for partial image data or full images

**DOI:** 10.1088/1361-6560/aa6070

**Published:** 2017-05-05

**Authors:** Jamie R McClelland, Marc Modat, Simon Arridge, Helen Grimes, Derek D’Souza, David Thomas, Dylan O’ Connell, Daniel A Low, Evangelia Kaza, David J Collins, Martin O Leach, David J Hawkes

**Affiliations:** 1 Centre for Medical Image Computing, Department of Medical Physics and Biomedical Engineering, University College London, Gower Street, London, WC1E 6BT, United Kingdom; 2 Translational Imaging Group, Centre for Medical Image Computing, Department of Medical Physics and Biomedical Engineering, University College London, Gower Street, London, WC1E 6BT, United Kingdom; 3 Radiotherapy Physics Department, University College London Hospitals NHS FT, Euston Road, London, NW1 2PG, United Kingdom; 4 Department of Radiation Oncology, University of Colorado School of Medicine, 1665 Aurora Court, Suite 1032 MS F706—Aurora, CO 80045, United States of America; 5 Department of Radiation Oncology, University of California Los Angeles, 200 Medical Plaza Way, Suite B265, Los Angeles, CA 90095, United States of America; 6 CRUK Cancer Imaging Centre, Institute of Cancer Research and Royal Marsden Hospital, 123 Old Brompton Road, London, SW7 3RP, United Kingdom; j.mcclelland@ucl.ac.uk

**Keywords:** respiratory motion modelling, image registration, respiratory surrogate signals, motion compensated image reconstruction, super-resolution, CT, MR

## Abstract

Surrogate-driven respiratory motion models relate the motion of the internal anatomy to easily acquired respiratory surrogate signals, such as the motion of the skin surface. They are usually built by first using image registration to determine the motion from a number of dynamic images, and then fitting a correspondence model relating the motion to the surrogate signals. In this paper we present a generalized framework that unifies the image registration and correspondence model fitting into a single optimization. This allows the use of ‘partial’ imaging data, such as individual slices, projections, or *k*-space data, where it would not be possible to determine the motion from an individual frame of data. Motion compensated image reconstruction can also be incorporated using an iterative approach, so that both the motion and a motion-free image can be estimated from the partial image data. The framework has been applied to real 4DCT, Cine CT, multi-slice CT, and multi-slice MR data, as well as simulated datasets from a computer phantom. This includes the use of a super-resolution reconstruction method for the multi-slice MR data. Good results were obtained for all datasets, including quantitative results for the 4DCT and phantom datasets where the ground truth motion was known or could be estimated.

## 1. Introduction

Respiratory motion is often a problem when acquiring images and planning and guiding interventions (e.g. radiotherapy) in the abdomen and thorax. It causes artefacts in reconstructed images, limiting their utility, and can cause misalignment between the intervention plan and the moving anatomy, limiting accuracy and leading to uncertainties in the delivered treatment. If the motion is known during the acquisition/intervention then it can be corrected for, e.g. using Motion Compensated Image Reconstruction, MCIR (Batchelor *et al* 2005, Rit *et al* 2009), or using gating or tracking when delivering treatment (Keall *et al* 2006). However, it is usually very difficult to directly measure the full motion during the acquisition/intervention.

One solution, which has been proposed for a wide range of applications, is using surrogate-driven respiratory motion models (McClelland *et al* 2013). These relate the internal motion to one or more respiratory surrogate signals, such as the displacement of the skin surface (McClelland *et al* 2011) or spirometry (Low *et al* 2005), which can be easily measured during the acquisition/intervention. A correspondence model describes the mathematical relationship between the surrogate signals and the internal motion, and is fitted using data acquired before the start of the acquisition/intervention. The model can then be used to estimate the internal motion from the surrogate signals during the acquisition/intervention.

**Figure 1.**
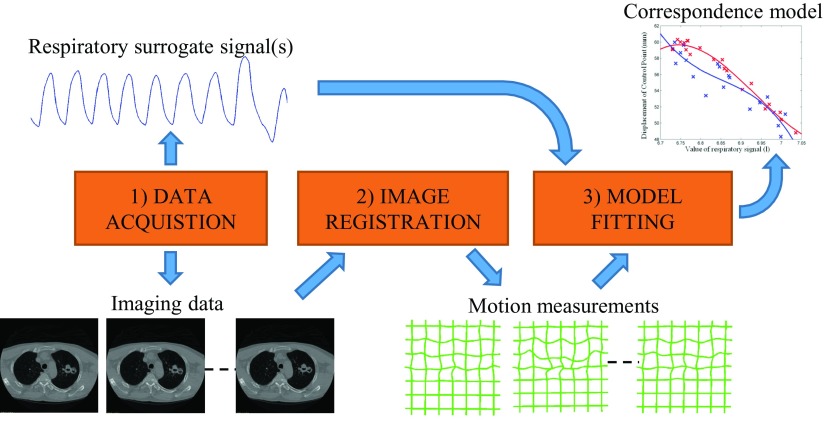
Typical approach for building a respiratory motion model. (1) Respiratory surrogate signals are acquired simultaneously with imaging data. (2) Image registration is used to determine the motion from the imaging data. (3) A correspondence model is fitted relating the motion to the surrogate signals.

Figure 1 illustrates how a motion model is typically built. Respiratory surrogate signals are simultaneously acquired with imaging data that capture the motion of interest. Then, image registration or a similar technique is used to determine the motion from the imaging data. Finally, a correspondence model is fitted relating the motion to the surrogate signals. This process is described in detail in McClelland *et al* (2013), along with the methods that have been used for each stage in the literature.

One problem with this approach, that has so far limited its practical applicability, is the difficulty in acquiring suitable imaging data for determining the full motion of interest. For most applications it is desirable to know the 3D motion of the anatomy, thus requiring 4D data, i.e. 3D  +  time. Many 4D imaging methods have been presented in the literature, including 4D-CT (Pan 2005), 4D-CBCT (Sonke *et al* 2005), and 4D-MR methods (von Siebenthal *et al* 2007, Yang *et al* 2015). However, most of these are respiratory-sorted (sometimes referred to as respiratory-correlated) methods. Due to scanner technology limitations it is impossible to acquire all the data required to form a full 3D volume fast enough to image the respiratory motion. Therefore, individual frames of ‘partial image data’ are acquired over several respiratory cycles, and are then sorted using either surrogate signals or image similarity measures (Paganelli *et al* 2015) to form ‘coherent’ volumes. The partial image data could be small slabs consisting of a few slices (Pan 2005), individual slices (von Siebenthal *et al* 2007), projection data (Sonke *et al* 2005), or lines of *k*-space (Rank *et al* 2016). Most of these methods assume reproducible motion during every respiratory cycle, so cannot be used to study or model breath-to-breath variations, and often contain artefacts caused by these variations. Some 4D-MR methods try to image breath-to-breath variability by acquiring each frame of data over many respiratory cycles (von Siebenthal *et al* 2007). However, these methods require long acquisition times, and as the images are formed from data acquired during different breath cycles they may not give a good representation of the true motion and its variability.

Furthermore, the different frames used to form the respiratory-sorted volumes often do not have exactly the same measured surrogate signal values. Such volumes are not well suited for building motion models as they will not have unique surrogate signal values for each volume. Some researchers have derived surrogate signals from the volumes (Fayad *et al* 2011), but then the motion and surrogate signals may be determined from different parts of the image data acquired during different respiratory cycles, and may not reflect the true relationship between surrogate and motion.

‘True’ 4D-MR methods, which acquire a full volume fast enough to image the respiratory motion, have also been proposed (Yang *et al* 2015). However, using current technology these methods suffer from poor image quality due to the trade-offs between spatial resolution, spatial coverage, and temporal resolution, limiting their use for respiratory motion modelling and compensation.

In this paper we propose a new generalized framework unifying image registration and motion model fitting into a single optimization. In addition to being a more efficient and robust approach, this enables the model to be fitted directly to the frames of partial image data, and does not require the data to be first sorted into full 3D volumes as described above, which would be required if building a surrogate-driven respiratory motion model using the typical approach. As the model is fitted to all of the frames of partial image data simultaneously the full 3D motion can be estimated, which can be challenging or impossible when using individual frames to estimate the motion. MCIR can also be easily incorporated into this framework using an iterative scheme. Consequently, the framework is particularly well suited to MCIR applications, as the motion model can be fitted directly to the unreconstructed partial image data, rather than requiring full reconstructed 3D images for fitting the model.

Some papers have proposed similar approaches to the framework presented here: Odille *et al* (2008) presented a method for fitting a linear motion model to MR *k*-space data; Hinkle *et al* (2012) presented a method for fitting a piece-wise linear model to CT projection or slice data; and Martin *et al* (2013) and Liu *et al* (2015) have presented methods for fitting linear models to CBCT projection data (modelling just tumour translations and deformations of the full anatomy respectively). However, these methods have been for specific types of data, registration algorithms, and motion models, whereas the framework presented here is generalized and applicable to a wide range of data, registration algorithms, and motion models. This work extends a previous conference publication (McClelland *et al* 2014) by giving a more detailed and rigorous presentation of the theory, using a more efficient B-spline based implementation, incorporating a super-resolution MCIR method, and presenting results from several real clinical datasets as well as phantom data.

## 2. Theory and methaodology

### 2.1. Respiratory motion models

To build a respiratory motion model image data are simultaneously acquired with one or more surrogate signals (figure 1). The image data consist of *N*_*i*_ dynamic images, ${{\mathbf{I}}_{1}}\cdots {{\mathbf{I}}_{{{N}_{i}}}}$, each acquired at a different time-point, *t*, and representing a different respiratory state. These should cover at least one respiratory cycle, although sometimes several cycles will be imaged so that inter-cycle variations can be sampled. A reference-state image, **I**_0_, is also required. This could be one of the dynamic images or it could be another image, e.g. a high quality breath-hold image.

Following the typical approach to building motion models shown in figure 1, the respiratory motion is first determined form the images, and then a correspondence model it fitted relating the motion and the surrogate signals. To determine the motion **I**_0_ is registered to each of the dynamic images, **I**_*t*_. Each registration is usually performed independently, and results in a spatial transformation parameterized by the motion parameters, $~{{\mathbf{M}}_{t}}=\left[{{m}_{t,1}},\cdots,{{m}_{t,{{N}_{\text{m}}}}}\right]$, where *N*_m_ is the number of motion parameters. e.g. **M**_*t*_ could be the parameters of an affine transform, a B-spline control point grid, or a voxel-wise deformation field.

The correspondence model relates **M**_*t*_ to the surrogate signals, ${{\mathbf{S}}_{t}}=\left[{{s}_{t,1}},\cdots,{{s}_{t,{{N}_{\text{s}}}}}\right]$, where *N*_s_ is the number of surrogate signals measured at each time-point, *t*. The model is parameterized by the model parameters, $\mathbf{R}\,={{\mathbf{R}}_{1}}\cdots {{\mathbf{R}}_{{{N}_{r}}}}$, where *N*_r_ is the number of model parameters per motion parameter, and ${{\mathbf{R}}_{n}}=\left[{{r}_{1,n}},\cdots,{{r}_{{{N}_{\text{m}}},n}}\right]$, i.e. the total number of model parameters is *N*_r_  ×  *N*_m_. The correspondence model can be represented as a function, *F*, of **S**_*t*_ and **R** that results in an estimate of the motion, ${{\mathbf{M}}_{{{F}_{t}}}}$:

\begin{eqnarray*}{{\mathbf{M}}_{{{F}_{t}}}}=F\left({{\mathbf{S}}_{t}},\boldsymbol{}~\mathbf{R}\right)\end{eqnarray*}

The correspondence model is fitted using a standard technique such as ordinary least squares, which minimizes the squared difference between the motion estimated by the model and the registration results over all time-points:

\begin{eqnarray*}\underset{\mathbf{R}}{{\min}}\,\underset{t}{\sum}\,\|{{\mathbf{M}}_{t}}-{{\mathbf{M}}_{{{F}_{t}}}}\|_{2}^{2}\end{eqnarray*}

### 2.2. Unifying image registration and respiratory motion modelling

The framework presented in this paper does not follow the typical approach described above. Instead, it directly optimizes the correspondence model parameters, **R**, on all the dynamic images simultaneously, such that the motion calculated using the correspondence model transforms **I**_0_ to best match the dynamic image data, i.e. ${{\mathbf{M}}_{t}}={{\mathbf{M}}_{{{F}_{t}}}}$.

Most image registration algorithms try to find the optimal values of **M**_*t*_ by minimising a cost function, *C*_*t*_, which consists of a (dis)similarity term, *Sim*, and an (optional) constraint term, *Con*.

\begin{eqnarray*}{{C}_{t}}\left({{\mathbf{I}}_{t}},{{\mathbf{I}}_{0}},{{\mathbf{M}}_{t}}\right)=Sim\left({{\mathbf{I}}_{t}},T\left({{\mathbf{I}}_{0}},{{\mathbf{M}}_{t}}\right)\right)+Con\left({{\mathbf{M}}_{t}}\right)\end{eqnarray*}

where **I**_*t*_ is the fixed (target) image, **I**_0_ the moving (source) image, and *T* the function that applies the transformation parameterized by **M**_*t*_ to image **I**_0_. Note, **I**_0_ is used as the moving image here as this is more convenient when using partial image data (see section 2.3). A common approach for optimizing the values of **M**_*t*_ is to estimate the gradient of *C*_*t*_ with respect to **M**_*t*_, i.e. $\frac{\partial {{C}_{t}}}{\partial {{\mathbf{M}}_{t}}}$, and then use a gradient based optimization such as gradient descent or conjugate gradient.

To directly optimize the correspondence model parameters on the image data the gradient of the cost function with respect to the model parameters, $\frac{\partial {{C}_{t}}}{\partial \mathbf{R}}$, is required. If the correspondence model can be differentiated to give the gradient of the motion parameters with respect to the model parameters, $\frac{\partial {{\mathbf{M}}_{t}}}{\partial \mathbf{R}}$, the chain rule can be applied, i.e.:

\begin{eqnarray*}\frac{\partial {{C}_{t}}}{\partial \mathbf{R}}=\frac{\partial {{\mathbf{M}}_{t}}}{\partial \mathbf{R}}\frac{\partial {{C}_{t}}}{\partial {{\mathbf{M}}_{t}}}\end{eqnarray*}

Therefore, any correspondence model can be used in this framework as long as it is possible to calculate $\frac{\partial {{\mathbf{M}}_{t}}}{\partial \mathbf{R}}$. This includes all of the popular correspondence models that have been used in the literature, such as linear, polynomial, and B-spline models (McClelland *et al* 2013). e.g. for a 2nd order polynomial model with one surrogate signal:

\begin{eqnarray*}{{\mathbf{M}}_{t}}={{\mathbf{R}}_{1}}s_{t}^{2}+{{\mathbf{R}}_{2}}{{s}_{t}}+{{\mathbf{R}}_{3}}\end{eqnarray*}

\begin{eqnarray*}\frac{\partial {{\mathbf{M}}_{t}}}{\partial {{\mathbf{R}}_{1}}}=s_{t}^{2},\boldsymbol{~}\frac{\partial {{\mathbf{M}}_{t}}}{\partial {{\mathbf{R}}_{2}}}={{s}_{t}},\boldsymbol{~}\frac{\partial {{\mathbf{M}}_{t}}}{\partial {{\mathbf{R}}_{3}}}=1\end{eqnarray*}

The correspondence model parameters are optimized on all of the image data by summing the cost function and gradient from each of the individual images:

\begin{eqnarray*}{{C}_{\text{total}}}=\underset{t=1}{\overset{{{N}_{i}}}{\sum}}\,{{C}_{t}},\quad \frac{\partial {{C}_{\text{total}}}}{\partial \mathbf{R}}=\underset{t=1}{\overset{{{N}_{i}}}{\sum}}\,\frac{\partial {{C}_{t}}}{\partial \mathbf{R}}\end{eqnarray*}

*C*_total_ and $\frac{\partial {{C}_{\text{total}}}}{\partial \mathbf{R}}$ can then be used to find the optimal values of **R** using the same gradient based optimization methods as used for standard registration between two images.

### 2.3. Using partial image data

With this new framework it is possible to fit the model directly to the partial image data, removing the need for full images, by modelling the image acquisition process:

\begin{eqnarray*}{{\mathbf{P}}_{t}}={{A}_{t}}\left({{\mathbf{I}}_{t}}\right)+\boldsymbol{\varepsilon }_{{t}}\end{eqnarray*}

where **P**_*t*_ is the measured partial image data at time *t*, **I**_*t*_ the (unknown) full image, and **ε**_*t*_ the imaging noise. *A*_*t*_ is the function which simulates the image acquisition, e.g. for projection data *A*_*t*_ would be the forward-projection operator and for slice data *A*_*t*_ would be the slice selection profile.

When fitting the correspondence model the partial image data is simulated from the transformed reference-state image, and the similarity term, *Sim*, measures the (dis)similarity between the measured and simulated partial image data, i.e.

\begin{eqnarray*}{{C}_{t}}\left({{\mathbf{P}}_{t}},{{\mathbf{I}}_{0}},{{\mathbf{M}}_{t}}\right)=Sim\left({{\mathbf{P}}_{t}},{{A}_{t}}\left(T\left({{\mathbf{I}}_{0}},{{\mathbf{M}}_{t}}\right)\right)\right)+Con\left({{\mathbf{M}}_{t}}\right)\end{eqnarray*}

However, *Sim*, is now defined in the space of the partial image data but the transformation parameterized by **M**_*t*_ is defined in the space of the full images. Therefore, when calculating $\frac{\partial {{C}_{t}}}{\partial {{\mathbf{M}}_{t}}}$ it is necessary to transform the gradient of *Sim* from partial image space into full image space. This is done using the adjoint of the image acquisition function, $A_{t}^{\ast}$, e.g. the adjoint of the forward projection operator is the back projection operator:

\begin{eqnarray*}\frac{\partial {{C}_{t}}}{\partial {{\mathbf{M}}_{t}}}=\frac{\partial Sim}{\partial {{\mathbf{M}}_{t}}}+\frac{\partial Con}{\partial {{\mathbf{M}}_{t}}}\end{eqnarray*}

\begin{eqnarray*}\frac{\partial Sim}{\partial {{\mathbf{M}}_{\boldsymbol{t}}}}=\frac{\partial {{\mathbf{I}}_{{{T}_{t}}}}}{\partial {{\mathbf{M}}_{\boldsymbol{t}}}}\frac{\partial Sim}{\partial {{\mathbf{I}}_{{{T}_{t}}}}}\end{eqnarray*}

\begin{eqnarray*}\frac{\partial Sim}{\partial {{\mathbf{I}}_{{{T}_{t}}}}}=A_{t}^{\ast}\left(\frac{\partial Sim}{\partial {{\mathbf{P}}_{{{A}_{t}}}}}\right)\end{eqnarray*}

where ${{\mathbf{I}}_{{{T}_{t}}}}=T\left({{\mathbf{I}}_{0}},{{\mathbf{M}}_{t}}\right)$, and ${{\mathbf{P}}_{{{A}_{t}}}}={{A}_{t}}\left({{\mathbf{I}}_{{{T}_{t}}}}\right)$. $\frac{\partial Sim}{\partial {{\mathbf{P}}_{{{A}_{t}}}}}$ is the gradient of the similarity measure with respect to the simulated partial image data, $\frac{\partial Sim}{\partial {{\mathbf{I}}_{{{T}_{t}}}}}$ is the gradient of the similarity measure with respect to the transformed reference-state image, and $\frac{\partial {{\mathbf{I}}_{{{T}_{t}}}}}{\partial {{\mathbf{M}}_{t}}}$ is the gradient of the transformed reference-state image with respect to the motion parameters, e.g. for B-spline transformations this is the spatial gradient of the reference-state image transformed by **M**_*t*_ and convolved with the B-spline kernel.

### 2.4. Incorporating motion compensated image reconstruction

The framework as described above assumes a reference-state image, **I**_0_, is available. However, if the motion is known MCIR can be used to reconstruct **I**_0_ from the partial image data (Batchelor *et al* 2005, Rit *et al* 2009, Van Reeth *et al* 2012), so a separate reference-state image is not required. MCIR can be incorporated into this framework using an iterative approach. Firstly, a MCIR is performed from the partial image data using an initial estimate of no motion (or using a previous model if one is available, e.g. from an earlier scan). The result will contain blurring and/or other motion artefacts, but can be used as an initial estimate of **I**_0_ to fit the model parameters, **R**, as described above. The fitted model is then used to perform another MCIR, updating the estimate of **I**_0_. The process continues to iterate between fitting **R** and reconstructing **I**_0_ until there is no more improvement or the maximum number of iterations is reached.

## 3. Experiments

### 3.1. Implementation details

The framework has been implemented using the open source NiftyReg^7^7http://cmictig.cs.ucl.ac.uk/research/software/niftyreg deformable image registration software (Modat *et al* 2010). This uses a B-spline transformation model and conjugate gradient optimisation in a multi-resolution approach. As all the experiments use intra-modality data, sum of squared differences (SSD) was used as the similarity measure. The use of a constraint term was found to be unnecessary.

Any correspondence model can be used which can be expressed as a linear combination of terms pre-calculated from the surrogate signal(s), ${{\varphi}_{n}}\left({{\mathbf{S}}_{t}}\right)$, i.e.

\begin{eqnarray*}{{\mathbf{M}}_{t}}=\underset{n=1}{\overset{{{N}_{r}}}{\sum}}\,{{\mathbf{R}}_{n}}{{\varphi}_{n}}\left({{\mathbf{S}}_{t}}\right)\end{eqnarray*}

then

\begin{eqnarray*}\frac{\partial {{\mathbf{M}}_{t}}}{\partial {{\mathbf{R}}_{n}}}={{\varphi}_{n}}\left({{\mathbf{S}}_{t}}\right)\end{eqnarray*}

This includes linear, polynomial, and B-spline models. e.g. for a linear model with two surrogate signals, ${{\mathbf{S}}_{t}}=\left[{{s}_{t,1}},{{s}_{t,2}}\right]$:

\begin{eqnarray*}{{\varphi}_{1}}\left({{\mathbf{S}}_{t}}\right)={{s}_{t,1}},\quad {{\varphi}_{2}}\left({{\mathbf{S}}_{t}}\right)={{s}_{t,2}},\quad {{\varphi}_{3}}\left({{\mathbf{S}}_{t}}\right)=1\end{eqnarray*}

for a 2nd order polynomial model with one surrogate signal, ${{\mathbf{S}}_{t}}={{s}_{t}}$:

\begin{eqnarray*}{{\varphi}_{1}}\left({{\mathbf{S}}_{t}}\right)=s_{t}^{2},\quad {{\varphi}_{2}}\left({{\mathbf{S}}_{t}}\right)={{s}_{t}},\quad {{\varphi}_{3}}\left({{\mathbf{S}}_{t}}\right)=1\end{eqnarray*}

and for a periodic B-spline model with 4 control points (*N*_r_  =  4) and respiratory phase as the surrogate signal, ${{\mathbf{S}}_{t}}={{\vartheta}_{t}}$ (where ${{\vartheta}_{t}}$ is the respiratory phase and has a value between 0 and 1):

\begin{eqnarray*}{{\varphi}_{n}}\left({{\mathbf{S}}_{t}}\right)={{B}_{i}}\left(\,j\right)\end{eqnarray*}

Where $i=\left(\lfloor 4{{\vartheta}_{t}}\rfloor +n-2\right)\,\bmod \,4$, $j=4{{\vartheta}_{t}}-\,\lfloor 4{{\vartheta}_{t}}\rfloor $, and ${{B}_{i}}$ is the *i*th cubic B-spline basis function:

\begin{eqnarray*}{{B}_{1}}\left(\,j\right)={{(1-j)}^{3}}/6\end{eqnarray*}

\begin{eqnarray*}{{B}_{2}}\left(\,j\right)=\left(3{{j}^{3}}-6{{j}^{2}}+4\right)/6\end{eqnarray*}

\begin{eqnarray*}{{B}_{3}}\left(\,j\right)=\left(-3{{j}^{3}}+3{{j}^{2}}+3j+1\right)/6\end{eqnarray*}

\begin{eqnarray*}{{B}_{4}}\left(\,j\right)={{j}^{3}}/6\end{eqnarray*}

The dynamic images can be full images, ‘slab’ images (consisting of a few slices), or individual slices. If the dynamic images are not full images the image acquisition function, *A*_*t*_, samples the deformed image, ${{\mathbf{S}}_{{{T}_{t}}}}$, at the slice(s) corresponding to the dynamic image, **P**_*t*_. The dynamic images can have a different resolution to the reference-state image, **I**_0_, enabling the use of a high resolution reference-state image with low resolution dynamic images, and permitting the use of super-resolution MCIR methods. If the dynamic images are lower resolution than **I**_0_ the function *A*_*t*_ first convolves the deformed image, $\boldsymbol{I}_{{{{T}_{t}}}}$ (which has the same resolution as **I**_0_), with an appropriate Gaussian kernel to account for the different image resolutions (Cardoso *et al* 2015), before sampling the slices corresponding to **P**_*t*_. Likewise, the adjoint function, $A_{t}^{\ast}$, first resamples $\frac{\partial Sim}{\partial {{\mathbf{P}}_{{{A}_{t}}}}}$ from the space of ${{\mathbf{P}}_{t}}$ into the space of the full deformed image, ${{\mathbf{I}}_{{{T}_{t}}}}$. If the dynamic images are lower resolution, the result is then convolved with the same Gaussian kernel as above.

Two MCIR methods have been implemented: a simple averaging of the deformed dynamic images, and a super-resolution reconstruction using the iterative back-projection method (Irani and Peleg 1991). When performing a MCIR the the dynamic image data (after applying $A_{t}^{\ast}$, i.e. $A_{t}^{\ast}\left({{\mathbf{P}}_{t}}\right)$) must be deformed into the space of **I**_0_. This could be done by estimating the inverse of the transformations parameterized by **M**_*t*_, but this can be computationally expensive. Instead the adjoint function *T* ^*^ is used, i.e. ‘push-interpolation’ is used instead of the usual ‘pull-interpolation’. This can potentially lead to ‘holes’ (voxels with no intensity information) in the deformed images, but as long as the holes are not at the same location for all dynamic images they will not be present in the final MCIR. If holes are present in the final MCIR, $A_{t}^{\ast}\left({{\mathbf{P}}_{t}}\right)$ can be resampled at a higher resolution before applying T^*^. However, sampling $A_{t}^{\ast}\left({{\mathbf{P}}_{t}}\right)$ at the same resolution as **I**_0_ and using linear interpolation resulted in no holes in the MCIRs for all the experiments below.

### 3.2. Real datasets

Four real data sets have been used: full images (4DCT), slab images (Cine CT), thin slices (helical CT), and thick slices (multi-slice MR).

The full images dataset is one of the freely available DIR-Lab 4DCT datasets^8^8Dataset 5: www.dir-lab.com/4DCT5.html (Castillo *et al* 2009). This consists of 10 CT volumes with a resolution of 1.1  ×  1.1  ×  2.5 mm^3^, representing different phases of the respiratory cycle.

The slab image dataset uses Cine CT volumes (unsorted 4DCT data) acquired at University College Hospital, London, as the dynamic images. Each Cine CT volume consists of 8  ×  2.5 mm slices (0.98 mm  ×  0.98 mm in-slice resolution), i.e. each volume only covers 2 cm, but they are acquired from 17 contiguous couch-positions such that the entire lungs are covered. Each couch-position is imaged 11 times, sampling just over one breath cycle, giving a total of 187 dynamic images. The surrogate signal was acquired using the Varian RPM system.

The thin slice dataset consists of individual CT slices from fast helical scans acquired at UCLA (Thomas *et al* 2014). Each helical scan consists of 301 slices and takes ~5 s to acquire. The images were resampled with a resolution was 1  ×  1  ×  1 mm^3^. 25 helical scans were acquired in alternating directions while the patient was freely breathing, although only 10 scans were used here. Therefore, each individual slice is imaged 10 times, with the images corresponding to arbitrary points in different breath cycles, such that both the intra- and inter-cycle variations are sampled for each slice. The surrogate signal was acquired using a respiratory belt.

The thick slice dataset consists of multi-slice MRI data acquired at the Institute of Cancer Research, London. Sagittal multi-slice MR data were acquired from a volunteer during free-breathing, from 30 adjacent slices encompassing both lungs. Each slice was imaged 10 times. The in-slice resolution was 1.77 mm  ×  1.77 mm and the slices thickness was 10 mm. The acquisition time for each slice was approximately 0.2 s, so the full acquisition took approximately 1 min. This acquisition was then repeated 4 more times, with a 2 mm left-right shift between each acquisition, giving a total of 150 overlapping slices. The surrogate signal was acquired from an MR-compatible ABC device working passively as a spirometer (Kaza *et al* 2015).

The details of the experiments performed on the different datasets are given in table 1. For the full images dataset a periodic B-spline correspondence model was used with respiratory phase as the surrogate signal. This can model intra-cycle variation (hysteresis), but not inter-cycle variation. For the other datasets a linear correspondence model was used, relating the motion to both the value and temporal derivative of the surrogate signal, i.e. ${{\mathbf{S}}_{t}}=\left[{{s}_{t}},{{\overset{\centerdot}{{s}}\,}_{t}}\right]$. This correspondence model has been popular in the literature due to its simplicity and ability to model both intra- and inter-cycle variations. The control point grid (CPG) spacing of the B-spline transformation was empirically set for each experiment, such that reasonable results were obtained. In all cases, the same spacing was used in all directions.

**Table 1.** Details of the different experiments performed on the real datasets.

Dataset	Exp. num.	Correspondence model	B-spline CPG spacing	Reference-state image	Segmented lungs?
Full images	1	Periodic B-spline	10 mm	End-exh. 4DCT	Yes
Slab images	1	Linear	10 mm	End-exh. 4DCT	Only in ref.
Slab images	2	Linear	5 voxels	MCIR: averaging	No
Thin slices	1	Linear	5 mm	Helical scan	No
Thin slices	2	Linear	5 mm	MCIR: averaging	No
Thick slices	1	Linear	30 mm	MCIR: averaging	Yes
Thick slices	2	Linear	30 mm	MCIR: super-res.	Yes

For experiment 1 on the full images and slab images datasets the end-exhalation 4DCT volumes was used as **I**_0_, as this is considered the most reproducible position and so the images usually contain the fewest artefacts. For experiment 1 on the thin slice dataset one of the helical scans was used as **I**_0_. As this was acquired while the patient was freely breathing the anatomy appears distorted, but due to the fast scan time there were no obvious missing or repeated structures. For the experiments that used the simple averaging MCIR method **I**_0_ was reconstructed with the same resolution as the dynamic images. For experiment 2 on the thick slice dataset the super-resolution MCIR method was used to reconstruct **I**_0_ with a voxel size of 2  ×  1.77  ×  1.77 mm^3^.

Note, as the surrogate signals have been normalized so that their mean values equal 0 the reference-state images reconstructed by MCIR will represent the average position of the anatomy during the acquisition. For the experiments that did not perform MCIR, **I**_0_ does not represent the average position of the anatomy. Therefore, and extra constant offset model parameter was included in the linear models to account for the difference between the **I**_0_ and the average position of the anatomy (this is not necessary for the B-spline model).

One problem encountered with several of the datasets was sliding motion between the lungs and the chest wall, which cannot be correctly modelled by a standard B-spline transformation. To alleviate this problem, the lungs were automatically segmented in some datasets using thresholding and morphological operations, as indicated in table 1. Voxels outside the lungs were set to the same intensity as soft tissue. For the slab images automatic lung segmentation was challenging in the dynamic images. Therefore, for experiment 1 the lungs were only segmented in **I**_0_, and for experiment 2, which used MCIR, they were not segmented at all. For the thin slice data good results were obtained with unsegmented images, so segmentation was not used.

For the full images manually located landmark points are available which can be used to quantitatively assess the results. These consist of 300 landmarks well distributed over both lungs, which have been located in the end-inhalation and end-exhalation images. A subset of 75 of the landmarks have also been located on all of the exhalation images. Both sets of landmarks were used to assess the fitted motion model using the ‘snap-to-voxel’ approach described in Castillo *et al* (2009). For comparison the landmark errors have also been calculated using an estimate of no motion, i.e. measuring the magnitude of the motion in the original images.

For the other datasets it is difficult to accurately locate landmarks in the images, therefore only qualitative assessments of their results have been possible.

### 3.3. Phantom datasets

**Figure 2.**
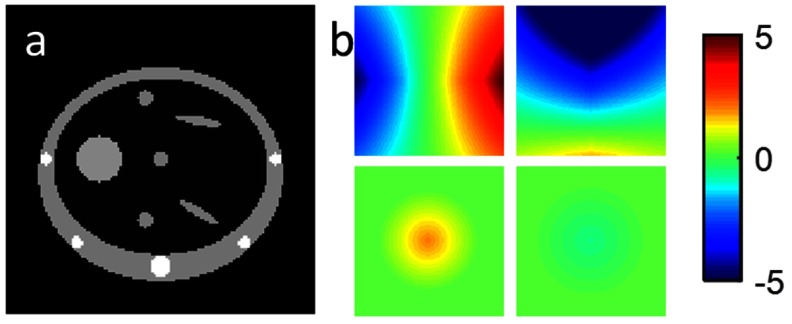
(a) ‘Lung-like’ 2D computer phantom. (b) The values of the correspondence model parameters used to animate the phantom: top-left  =  *r*_*x*,1_, top-right  =  *r*_*y*,1_, bottom-left  =  *r*_*x*,2_, bottom-right  =  *r*_*y*,2_.

As it is difficult to estimate the ground truth motion for most of the real datasets, additional experiments were performed using datasets generated from a computer phantom. For these experiments the ground truth motion and reference-state image, **I**_true_, are known, so the results of the motion model fitting and the MCIR can be quantitatively assessed. A simple 2D ‘lung-like’ phantom was used for all the experiments (figure 2(a)). The phantom was animated using displacement fields generated from a linear correspondence model relating the motion to a surrogate signal and its temporal gradient. For the slab images, thin slices, and thick slices, the surrogate signals from the real datasets were used to animate the phantom. For the full images a real surrogate signal is not available, so an idealized surrogate signal from a cos^4^ function was used. For all of the phantom image datasets Gaussian noise with a standard deviation of 3% of the maximum image intensity (1500) was added to the simulated images. When animating the phantom, the displacement field itself was used as the motion parameters, **M**_*t*_, whereas the B-spline control point displacements are used as **M**_*t*_ when fitting the model. This means there are two motion parameters at each pixel, the displacement in the x and y directions, and therefore four correspondence model parameters for each pixel, *r*_*x*,1_, *r*_*x*,2_, *r*_*y*,1_, and *r*_*y*,2_. The values of these model parameters were manually defined such that they were smoothly varying over the phantom (figure 2(b)), and produced plausible looking motion that included deforming anatomy and intra- and inter-cycle variations.

The phantom datasets and the experiments performed with them mimicked the real datasets and experiments as closely as possible. The same number of images/slabs/slices was used as for the real data, but as the phantom was only 2D, images were 2D, and slices were 1D. Different sized phantoms were required for the different datasets: 128  ×  128 pixels for the full images, 136  ×  136 pixels for the slab images, 301  ×  301 pixels for the thin slices, and 150  ×  150 pixels for the thick slices. However, the phantom was resized so that its proportions were always the same. As the phantom used for the thin slices had approximately twice as many pixels as for the other datasets, the magnitude of motion was twice as large (in pixels) for this dataset. The thick slices were simulated by convolving the deformed images with a Gaussian kernel representing a slice thickness of 5 pixels. The surrogate signals from the real datasets were also used for the phantom experiments, and the same correspondence models and MCIR methods were applied as for the real data. The B-spline CPG spacing was empirically set to 10 pixels for the thin slice dataset, and to 5 pixels for all the others. As the phantom datasets did not contain sliding motion there was no need for segmentation.

The results of all the phantom experiments were assessed by calculating the Displacement Field Error, DFE, defined as the 2D Euclidean distance at each pixel between the true displacement field and the displacement field resulting from the fitted motion model. The DFE was calculated at every pixel inside the deformed phantom for every time-point. Pixels outside the phantom were ignored as they do not contain any image data to guide the model fitting. The DFE was also calculated for each experiment using an estimate of no motion, to quantify the amount of motion present in the original data. The experiments that performed MCIRs were also assessed by comparing the reconstructed **I**_0_ to **I**_true_. This was accomplished by calculating the absolute difference in the image intensities at every pixel and the correlation coefficient between the images. Similar calculations were performed for an image reconstructed using the same method (averaging or super-resolution) but assuming no motion.

## 4. Results

### 4.1. Real datasets

Videos showing the results of the experiments on the real datasets are available as supplementary files. These show the original dynamic images, **I**_*i*_ or **P**_*i*_, the simulated dynamic images, ${{\mathbf{I}}_{{{T}_{i}}}}$ or ${{\mathbf{P}}_{{{A}_{t}}}}$, and a colour overlay of these. In addition, for the datasets using partial image data (i.e. the slab and slice datasets), the videos also show one or more views through the full deformed reference-state image, ${{\mathbf{I}}_{{{T}_{i}}}}$.

#### 4.1.1. Full images.

As shown in the supplementary movie (real_data_full_images.mp4) (stacks.iop.org/PMB/62/4273/mmedia), there is generally a good match between **I**_*i*_ and ${{\mathbf{I}}_{{{T}_{i}}}}$. Some mismatches are noticeable near the back of the lungs due to sliding motion, even though the lungs had been segmented. There are also some smaller mismatches throughout the lungs, but some of these are due to artefacts present in the original dynamic 4DCT images. Some images contain blurred and repeated structures (due to the CT rotation time being too slow to ‘freeze’ the motion) but the ${{\mathbf{I}}_{{{T}_{i}}}}$ images do not contain these artefacts as they are not present in **I**_0_. Also, the motion seen in the original 4DCT images appears ‘wobbly’ due to binning errors and inter-cycle variations, but the motion model estimates smooth and continuous motion over the whole breath cycle. The landmark errors given in table 2 support the qualitative results observed in the movie. Landmark errors are greatly reduced when using the fitted motion model, and are comparable to those reported on the DIR-Lab website^9^9Dataset 5: www.dir-lab.com/Results.html, where the mean errors range from 1.07 mm to 3.59 mm. Some of the more recent methods have lower landmark errors than achieved here, but this may be partly due to most other methods registering the images individually, which can result in lower landmark errors but will also reproduce the ‘wobbly’ motion seen in the 4DCT.

**Table 2.** Landmark errors, in mm, for the full images dataset using an estimate of no motion and using the fitted motion model to estimate the motion (model). Landmark set 1 are the 300 landmarks identified in the end-exhalation and end-inhalation images only. Landmark set 2 are the 75 landmarks that have been identified in all exhalation images.

	Landmark set 1		
	Mean	Std. dev.	95th percentile
No motion	7.48	5.51	17.67
Model	1.88	2.03	5.24

	Landmark set 2		
	Mean	Std. dev.	95th percentile
No motion	3.57	4.49	15.04
Model	1.72	2.25	4.28

#### 4.1.2. Slab images.

There was a fairly good match between **P**_*i*_ and ${{\mathbf{P}}_{{{A}_{t}}}}$ for both experiments, as can be seen in the supplementary movies (real_data_slab_images_exp1.mp4 and real_data_slab_images_exp2.mp4). As **P**_*i*_ only sample one respiratory cycle at each couch-position it is difficult to assess how accurately the models estimate the inter-cycle variations, but the images of ${{\mathbf{I}}_{{{T}_{i}}}}$ show plausible motion over the entire field of view, exhibiting both intra- and inter-cycle variations. As with the 4DCT data, there are some noticeable mismatches at the back of the lungs due to the sliding motion, and some smaller mismatches in other regions, but again these are partly due to blurred and repeated structures in some of the Cine CT volumes.

**Figure 3.**
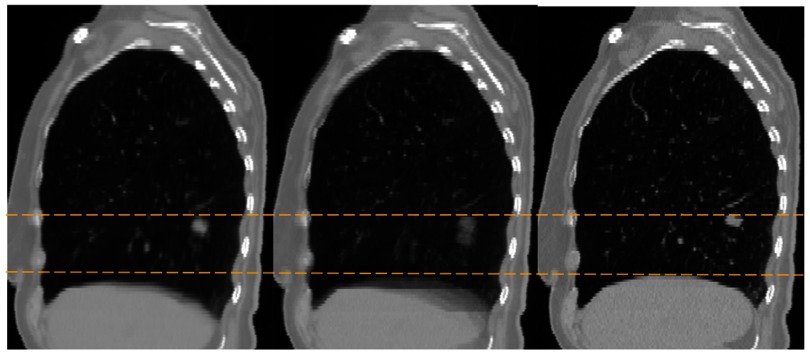
The MCIR produced from the real slab images dataset (left), a similar image formed without applying any motion correction (middle), and the end-exhalation 4DCT volume for the same subject (right). The dashed lines represent the height of the tumour and diaphragm in the end-exhalation 4DCT volume, and show that these are both lower in the MCIR. This is expected as the MCIR corresponds to the average position of the anatomy.

Figure 3 compares the end exhalation 4DCT volume with the MCIR produced in experiment 2, and a similar image formed without applying any motion correction (i.e. the result of averaging all of the Cine CT volumes together). It is evident that blurred structures are greatly reduced in the MCIR compared to the result with no motion correction, indicating that most of the motion has been well recovered. Some structures still appear slightly blurred compared to the 4DCT volume, particularly near the back of the lungs where sliding motion occurs. This blurring is partly caused by errors in the estimated motion, but also due to the structures appearing blurred in some of the Cine CT volumes.

#### 4.1.3. Thin slices.

**Figure 4.**
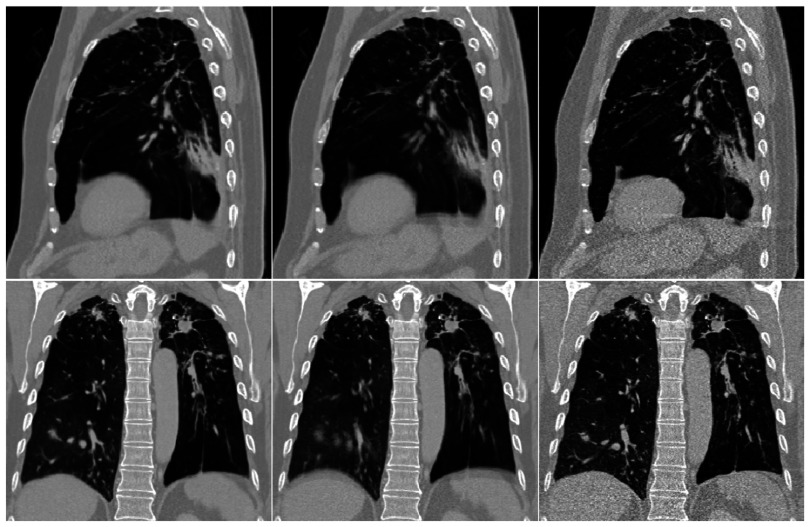
Sagittal (top) and Coronal (bottom) views through the MCIR produced from the real thin slices dataset (left), a similar image formed without applying any motion correction (middle), and one of the fast helical scans from the same subject (right).

For both experiments there is a very good match between **P**_*i*_ and ${{\mathbf{P}}_{{{A}_{t}}}}$, as can be seen in the supplementary movies (real_data_thin_slices_exp1.mp4 and real_data_thin_slices_exp2.mp4). The images of ${{\mathbf{I}}_{{{T}_{i}}}}$ show plausible motion, including intra- and inter-cycle variations, over the entire field of view, and even seem to reproduce most of the sliding motion seen at the back of the lung. Since **P**_*i*_ sample both the intra- and inter-cycle variations at each location, this implies the true variations have been modelled well. Figure 4 shows the helical scan used as **I**_0_ for experiment 1, the MCIR produced by experiment 2, and a similar image formed without applying any motion correction. The blurring has been almost completely removed in the MCIR, with the majority of structures appearing as sharp as in the helical scan, indicating the motion has been modelled very well. In addition, the signal-to-noise ratio was noticeably improved in the MCIR due to combining the image data from all 10 slices at each location.

#### 4.1.4. Thick slices.

${{\mathbf{P}}_{{{A}_{t}}}}$ match **P**_*i*_ well for both experiments, as can be seen in the supplementary movies (real_data_thick_slices_exp1.mp4 and real_data_thick_slices_exp2.mp4). Similar to the thin slices dataset, **P**_*i*_ sample both the intra- and inter-cycle variations at each location, which indicates these have been modelled well. The images of ${{\mathbf{I}}_{{{T}_{i}}}}$ show plausible motion over the entire field of view, although the motion at the edge of the lungs looks questionable, particularly for experiment 2. This is due to the sliding motion between the lungs and the ribs, and the ribs sometimes being included in the segmented lungs as they have similar intensity in the original MR images.

**Figure 5.**
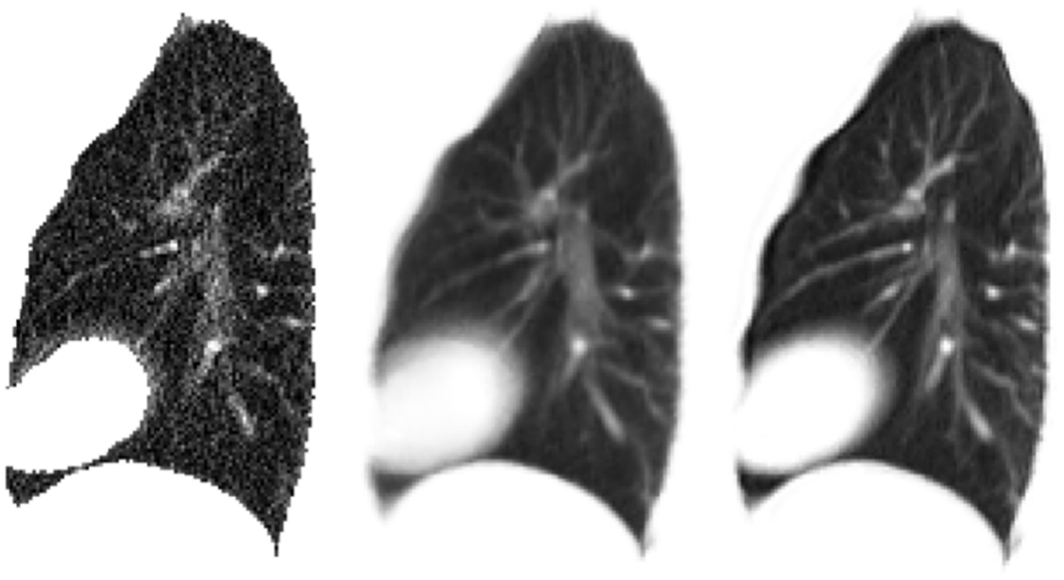
One of the original dynamic images from the real thick slice dataset (left), and the corresponding simulated dynamic images from experiment 1 (middle) and experiment 2 (right).

**Figure 6.**
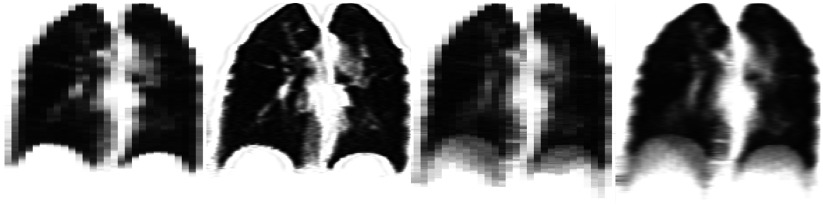
The MCIRs produced from the real thick slice dataset in experiment 1 using the averaging method (far-left) and experiment 2 using the super-resolution method (middle-left), and similar images formed using the averaging method (middle-right) and the super-resolution method (far-right) without applying motion correction.

Figure 5 shows an example **P**_*i*_ (after lung segmentation), together with the corresponding ${{\mathbf{P}}_{{{A}_{t}}}}$ from both experiments. The signal-to-noise ratio has greatly improved in ${{\mathbf{P}}_{{{A}_{t}}}}$ from experiment 1, due to averaging the dynamic images, and ${{\mathbf{P}}_{{{A}_{t}}}}$ from experiment 2 shows even finer structure inside the lung due to the super-resolution reconstruction and an increased number of dynamic images. Figure 6 shows coronal views through the MCIRs produced by both experiments, together with similar images formed without applying any motion correction. The blurring has been removed in the MCIRs, indicating that motion has been recovered well. Much more detail can be seen in the MCIR from experiment 2, indicating that the super-resolution method has been applied successfully and that the motion has been modelled well.

### 4.2. Phantom datasets

Videos showing the results of the experiments on the phantom datasets are available as supplementary files. Each of these show the original dynamic images, **I**_*i*_ or **P**_*i*_, the simulated dynamic images, ${{\mathbf{I}}_{{{T}_{i}}}}$ or ${{\mathbf{P}}_{{{A}_{t}}}}$, and a colour overlay of these. Also, for the phantom datasets using partial image data the true deformed reference-state images, **I**_*i*_, are shown together with the reference-state image deformed using the fitted model, ${{\mathbf{I}}_{{{T}_{i}}}}$, and a colour overlay of these. The videos show that there were good matches between **P**_*i*_ and ${{\mathbf{P}}_{{{A}_{t}}}}$ and between **I**_*i*_ and ${{\mathbf{I}}_{{{T}_{i}}}}$ for all phantom datasets experiments. In addition, for the experiments that performed MCIRs, **I**_0_ closely resemble **I**_true_, although for experiment 1 on the thick slice data **I**_0_ has a noticeably lower through-slice resolution, as expected.

The qualitative results shown in the videos are supported by the quantitative results in tables 3 and 4. Table 3 gives the Displacement Field Error (DFE) for each experiment. When using the fitted model the DFE is greatly reduced for all experiments, in most cases to below 1 pixel, indicating the motion has been recovered very well. The DFE is larger for experiment 1 on the thin slice data, but the magnitude of the motion was twice as large for this dataset (as it represents higher resolution data), and the relative reduction in DFE when using the model is comparable to the other datasets. Interestingly, the use of MCIR (i.e. experiment 2) leads to a considerably lower DFE for the thin slice dataset but not for the slab images dataset. For the thick slice data, experiment 2, which uses a super-resolution MCIR, yields a much lower DFE than experiment 1, which uses the averaging MCIR. This result is expected, as the super-resolution MCIR has a higher resolution and hence can recover the motion more accurately.

**Table 3.** Displacement field error (DFE), in pixels, for the different datasets and experiments, assuming no motion (no motion) and using the fitted models to estimate the motion (model).

Dataset	Experiment	DFE—no motion			DFE—model		
		Mean	Std. dev.	95th percentile	Mean	Std. dev.	95th percentile
Full Images	1	3.56	3.79	11.13	0.42	0.35	1.08

Slab Images	1	3.53	3.61	11.09	0.49	0.47	1.26
	2	2.82	2.19	7.15	0.53	0.66	1.94

Thin Slices	1	8.37	6.82	20.99	1.52	1.57	4.97
	2	5.98	3.82	12.92	0.49	0.64	1.87

Thick Slices	1	2.83	2.02	6.91	0.84	0.80	2.47
	2	2.98	1.93	6.56	0.39	0.48	1.38

**Table 4.** Absolute intensity differences and correlation coefficients between **I**_0_ and **I**_true_. Avg.  =  MCIR performed by averaging the deformed dynamic images, S-R  =  MCIR preformed using the super-resolution method, model  =  motion estimated using fitted motion model, no motion  =  MCIR performed assuming no motion.

Dataset	MCIR	Absolute intensity difference			Correlation coefficient
		Mean	Std. dev.	95th percentile	
Slab images	Avg.—model	23.78	55.93	156.03	0.99
	Avg.—no motion	97.85	180.73	538.29	0.83

Thin slices	Avg.—model	15.20	39.00	28.83	0.99
	Avg.—no motion	94.67	187.22	563.21	0.82

Thick slices	Avg.—model	63.66	120.42	365.63	0.93
	Avg.—no motion	101.49	170.14	484.51	0.85
	S-R—model	45.37	76.04	209.13	0.97
	S-R—no motion	101.65	175.56	515.81	0.83

**Figure 7.**
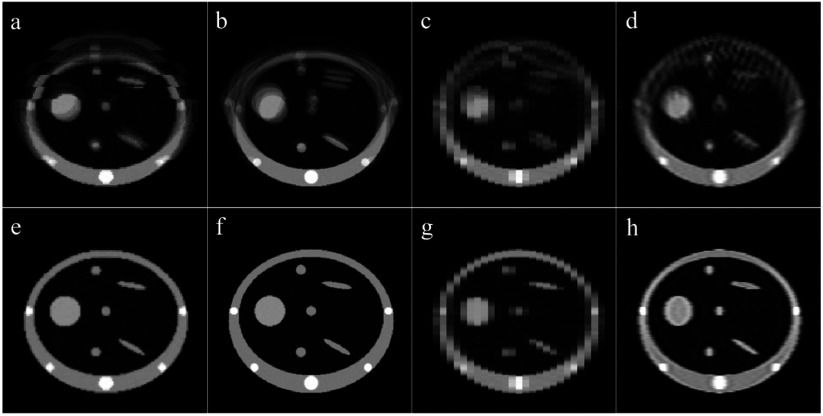
MCIRs generated from the phantom datasets assuming no motion (a)–(d) and using the fitted motion models to estimate the motion (e)–(h). The MCIRs were constructed by averaging the deformed dynamic images for the slab datasets (a) and (e), the thin slice dataset (b) and (f), and the thick slice dataset (c) and (g), and using the super-resolution method for the thick slice dataset (d) and (h).

Table 4 displays the results of comparing **I**_0_ to **I**_true_, and figure 7 shows images of **I**_0_ for each experiment performing MCIR. These results indicate that the motion has been recovered well by the fitted motion models and that **I**_0_ closely resembles **I**_true_ for all experiments. As expected, the results for the thick slice dataset are not as good as for the thin slice and slab image datasets, because the dynamic images have a lower resolution than **I**_true_. However, the use of the super-resolution method improves the results, and produces an **I**_0_ more similar to **I**_true_.

## 5. Discussion and conclusions

This paper has presented a generalized framework that unifies image registration and fitting a respiratory correspondence model into a single optimisation, and can incorporate motion compensated image reconstruction (MCIR) using an iterative scheme. This framework allows motion models to be fitted directly to the unsorted partial image data, as well as to respiratory-sorted 3D volumes. This overcomes the need for using respiratory-sorted volumes when building the surrogate-driven motion models, which are required by most other methods, and we believe has been a major factor limiting the accuracy and applicability of such methods to date.

There are a few other methods from the literature that could potentially be directly applied to the unsorted slab and slice data used in this paper. The method of Hinkle *et al* (2012) could be applied to any of the slab and slice datasets, however, their method uses a piece-wise linear motion model, and so cannot model inter-cycle variations. In McClelland *et al* (2011) a motion model is fitted directly to Cine CT slab data using the typical approach of first performing the image registrations and then fitting the motion model. However, the Cine CT data used in McClelland *et al* (2011) had 12 slices, whereas the data used here only has 8 slices, and the image registration results were often found to be unsatisfactory on the 8-slice Cine CT data. In addition, the method in McClelland *et al* (2011) is not appropriate for single slice data. In Thomas *et al* (2014) a motion model is fitted to the same thin slice CT data used in this paper, and the final results are similar to the results from this paper. However, the method of Thomas *et al* (2014) requires the helical scans to be acquired very quickly, so as to minimise the distortions and artefacts in the full 3D helical images, and hence allow them to be registered to each other. The method presented here does not require the 3D helical images, so can use data acquired more slowly on a standard CT scanner, as long as the individual slices are acquired fast enough to be considered motion-artefact free (a 0.5 s rotation time is usually considered fast enough, although even then there can still be artefacts, especially for slices acquired at mid in/exhale).

This framework has been implemented using the NiftyReg registration software, and has been applied to phantom and real datasets consisting of full images, small slabs, and individual slices. Good quantitative results were obtained on the phantom data, where the ground truth motion and true reference-state image were known, and on the real 4DCT dataset, where manually annotated landmark points are available. Promising results were also obtained on the other real datasets, but these could only be assessed qualitatively as it was difficult to accurately estimate the ground truth motion. Intra-cycle and inter-cycle variations appear to have been successfully modelled when present in the data. The results indicate that MCIR can be successfully incorporated into the framework, and that a super-resolution method can be used.

The results presented in this paper represent a general proof of principle. Future work is required to tailor the framework for specific applications. This will include investigating the best choices for the surrogate data, correspondence model, imaging data, registration algorithm, and MCIR method (if used), as well as tuning the various parameters settings. The choice of surrogate signals will depend on which signals can be easily acquired during the acquisition/intervention and how well they relate to the respiratory motion and its variation. The choice of correspondence model will depend on the type of motion and variation to be modelled, and on the surrogate signals and imaging data available. See McClelland *et al* (2013) for a detailed discussion of the choices available. The imaging data used in this paper had already been reconstructed into 2D slices or 3D volumes. Future work will investigate using ‘raw’ imaging data, such as CT projections or MR *k*-space data, instead of reconstructed images. Work will also be required to determine how much data to acquire and the exact protocol to use, so as to best capture the motion and variation to be modelled whilst limiting the scanning time and (for CT) dose.

The popular B-spline registration algorithm was used in this paper, as it has previously been shown to provide good results in a wide range of applications, and an efficient open source implementation was available. However, one of the drawbacks of this algorithm (and many others) is its inability to properly account for sliding motion which often occurs between the lungs and the chest wall during respiration. A number of approaches to handling sliding motion have been proposed (Delmon *et al* 2013) which could be incorporated into the framework presented in this paper. Some of these approaches require the sliding regions to be segmented in the images. Segmenting the sliding regions in the dynamic image data may be impossible if they are partial or raw image data, but it should be possible in the reference-state image. However, if this is formed using MCIR the segmentation will need repeating every time a new MCIR is performed. Future work will also investigate the use of a stationary velocity field transformation model, which is guaranteed to give diffeomorphic transformations (Wilms *et al* 2014), as well as different similarity measures, constraint terms, and optimisation methods.

Super-resolution reconstruction has previously been proposed for lung MR data (Van Reeth *et al* 2015), but this required 3D volumes to determine the motion so was based on the assumption that there was no inter-cycle variation. To the best of our knowledge, this is the first time that super-resolution reconstruction has been used directly on the slice data so that inter-cycle variations can be accounted for. The iterative back-projection method was used as this was the simplest to implement. Future work will investigate the use of different super-resolution methods (Van Reeth *et al* 2012), as well as other iterative and non-iterative MCIR methods applicable to ‘raw’ imaging data. Additionally, the possibility of jointly optimising the image and model parameters simultaneously, rather than iteratively, will be explored.

In addition to tailoring the framework to the specific applications, the results will need to be thoroughly validated. As highlighted in this paper, this can be challenging for partial image data, and will be even more challenging if raw imaging data are used in the future. It will require a combination of software and hardware phantoms, manually annotated clinical data (where possible), manually and automatically graded results, and extra data specifically for validation (e.g. motion traces from implanted markers).

The method presented here also has some inherent assumptions and limitations. Like all surrogate-driven motion modelling approaches, there is an assumption that the motion is related to some surrogate signal(s), and that the signal(s) can be easily acquired during the image acquisition (and if the models are to be used to guide treatment, during the treatment delivery). However, there is good evidence (e.g. from the Cyberknife system (Hoogeman *et al* 2009)) that this assumptions holds well, at least for short time frames. If partial imaging data is used, then the method will also be limited by how accurately the image acquisition function (and its adjoint) represents the real image acquisition, but this is a limitation for all image reconstruction methods. This is also related to the issue of computation time, as more accurate image acquisition functions may be more computationally expensive. But computation time is also affected by other factors, in particular the type and amount of data and the type of MCIR used (if any). e.g. experiments 1 and 2 on the real thick slice (MR) dataset took 6 and 107 min to run respectively, and experiments 1 and 2 on the real thin slice (CT) dataset took 297 and 386 min to run respectively. It should be noted that what is considered an acceptable computation time depends very much on the specific application that the model is being used for, and ensuring that the computation time is acceptable will be an important part of tailoring the framework for different applications.

In conclusion, this paper has presented a general purpose and versatile framework that can be used for building respiratory motion models from partial imaging data, as well as full images. Much work is still required to optimize and fully exploit the framework for different types of data and clinical applications, but very promising results have been obtained on several phantom and real datasets, and the framework has potential uses is a wide range of clinical applications.
